# Supplementary material for: Life Story Book to enhance communication in persons with dementia: A systematic review of reviews
Source: PLoS One. 2023 Oct 5;18(10):e0291620. doi: 10.1371/journal.pone.0291620 (PMC10553343; doi:10.1371/journal.pone.0291620)
Supplement: S1 Table — (DOCX) [file pone.0291620.s001.docx]

**S1 Table. Quality assessment ratings of review studies included (AMSTAR)**

| Author, year | AMSTAR SCORE | | | | | | | | | | | AMSTAR score Summary | | | |
| --- | --- | --- | --- | --- | --- | --- | --- | --- | --- | --- | --- | --- | --- | --- | --- |
|  | 1. Was an 'a priori' design provided? | 2. Was there duplicate study selection and data extraction? | 3. Was a comprehensive literature search performed? | 4. Was the status of publication (i.e. grey literature) used as an inclusion criterion? | 5. Was a list of studies (included and excluded) provided? | 6. Were the characteristics of the included studies provided? | 7. Was the scientific quality of the included studies assessed and documented? | 8. Was the scientific quality of the included studies used appropriately in formulating conclusions? | 9. Were the methods used to combine the findings of studies appropriate? | 10. Was the likelihood of publication bias? assessed? | 11. Was the conflict of interest included? | Yes | No | Cannot answer | Not applicable |
| Subramaniam & Woods, 2012 | Y | Y | Y | N | N | Y | CA | CA | Y | N | NA | 5 | 3 | 2 | 1 |
| Grøndahl et al., 2017 | Y | Y | Y | N | Y | Y | Y | Y | Y | N | NA | 8 | 2 | 0 | 1 |
| Elfrink et al., 2018 | Y | Y | Y | Y | N | N | Y | Y | Y | N | NA | 7 | 3 | 0 | 1 |
| Parker et al., 2020 | Y | Y | Y | N | Y | Y | N | N | N | N | NA | 5 | 5 | 0 | 1 |
| Doran et al., 2019 | Y | Y | Y | Y | Y | Y | Y | Y | Y | N | CA | 9 | 1 | 1 | 0 |

Y = Yes

N = No

CA = Cannot answer

NA = Not applicable
