# Supplementary material for: Life Story Book to enhance communication in persons with dementia: A systematic review of reviews
Source: PLoS One. 2023 Oct 5;18(10):e0291620. doi: 10.1371/journal.pone.0291620 (PMC10553343; doi:10.1371/journal.pone.0291620)
Supplement: S2 Table — (DOCX) [file pone.0291620.s002.docx]

**S2 Table. ENTREQ methodology quality assessment for qualitative review studies**

| Author, year | Introduction | | | | Literature search and selection of studies | | Appraisal and synthesis of findings | | | | | Total scores for Yes |
| --- | --- | --- | --- | --- | --- | --- | --- | --- | --- | --- | --- | --- |
|  | Aim: Was the research question clearly stated? | Search approach: Was the approach to searching for the literature appropriate for the research question? | Inclusion criteria: Were the inclusion/exclusion criteria clearly described? | Competence: Were there a sufficient number of researchers involved in the synthesis who had adequate competence? | Search strategy: Was the search strategy sufficient to capture the relevant literature? | Study screening: Was the selection of relevant studies conducted independently by more than one reviewer and with consensus? | Appraisal: Was risk of bias (or methodological quality) formally assessed using appropriate criteria? | Appraisal process: Was the appraisal conducted independently by more than one reviewer and with consensus? | Synthesis: Was the synthesis method appropriate for the research question? | Synthesis: Was the synthesis conducted appropriately? | Synthesis output: Were findings clearly grounded in the primary studies? |  |
| Moos et al., 2006 | N | Y | N | NI | Y | NI | NI | NI | N | Y | N | 3 |
| Kindell et al., 2014 | Y | Y | Y | Y | Y | Y | NI | NI | Y | Y | NI | 8 |

Y = Yes

N = No

NI = No Info
