# Supplementary material for: Life Story Book to enhance communication in persons with dementia: A systematic review of reviews
Source: PLoS One. 2023 Oct 5;18(10):e0291620. doi: 10.1371/journal.pone.0291620 (PMC10553343; doi:10.1371/journal.pone.0291620)
Supplement: Attachment — Submitted filename: Review - life Story Book.docx [file pone.0291620.s005.docx]

The approach to the literature review is sound, as it includes relevant approaches for the different study types selected.

While the conclusions are supported by the evidence presented, there are some issues that need addressing, as follows:

1. Evidence - unless there is definitive evidence that the life story book actually improves quality of care, interactions, communication, memory, quality of life etc., then these cannot be claimed. It appears that most of the evidence relied on is anecdotal, i.e. what the staff or family report from their own observation, rather than from clinical assessment or direct observation by a researcher. On Page 3, lines 118-120, i suggest the statement be changed to '...quality of life and other outcomes for the person living with dementia. Benefits may include improved cognition and mood, reduced depression...'. It is important not to overstate the benefits of the life story book.

Wherever terms such as 'significant' (Conclusion) are used, replace these with more suitable terms.

2. Grammar - there are many areas where incorrect grammar is used and this makes it hard to understand what message is conveyed, or it invalidates the statement. I have listed recommendations on use of correct grammar, as follows:

Abstract:

2nd sentence - replace 2nd sentence with 'This involves collecting the life stories and memories of the person living with dementia and compiling them into a book or folder, which is used by staff or family to assist the person recall these memories'.

3rd sentence - replace the 3rd sentence with 'Evidence on the use, benefits and influences of the life story book in dementia carte is limited.' Commence another sentence as 'This systematic literature review aimed to ...'.

Last sentence - This needs to be broken into two sentences, finishing at 'communication'. Commence a new sentence as 'Guidelines and training are also required to make the best use of the life story book'.

Page 2, line 92. Replace 'nurses that' with 'nurses who'.

Page 3, lines 98-99. Replace 'better delivery of care for the person with dementia that it becomes more person centred care as a result' with 'more person-centred care for persons living with dementia'.

Page 3, lines 118-120. Replace 'quality of care' with 'quality of life', as care quality does not support the following statement. i suggest the statement be changed to '...quality of life and other outcomes for the person living with dementia. Benefits may include improved cognition and mood, reduced depression...'. It is important not to overstate the benefits of the life story book.

Page 9, line 238. There are missing words at the beginning of the sentence, commencing '[14]'.

Page 17, line 246. Replace 'The life story book product used varied..' with 'Use of the life story book varied for ..'

Page 18, line 266. Replace '..three of the reviews reported studies used the...' with 'Three studies used the ...'.

Table 4. Grammatical errors need correcting.

Page 37, lines 339-341.

Rewrite the sentence as ‘This may be an issue, however, for the person living with dementia who has impaired memory and is unable to recall recent events or pleasurable times. These persons may not have the capacity to maintain a sense of continuity between events and memories’.

Page 37, line 344. Explain what is meant by ‘progress’.

Page 39, line 388. Remove ‘those’.

3. Terminology - many terms are used inappropriately, which alters the true meaning of what is being conveyed. I have listed recommended words to replace ones used incorrectly, as follows:

Page 2, line 91. Replace 'vastly implemented' with 'widely implemented'.

Page 2, line 95. Replace 'that states' with 'that indicates'.

Page 7, line 179. Replace 'reviews' with 'review'.

Page 8, line 199. Use a more suitable term to replace 'database hits'.

Page 37, line 342. Replace ‘process’ with ‘processes’.

Page 38, line 362. Replace ‘..that are of the best interest of the person..’ with ‘tahts are beneficial for the person living with dementia’.

Page 38, line 376. Replace ‘still scarce’ with ‘scant’.

Page 39, line 404. Explain what ‘managing’ the person living with dementia means. Should this be replaced with ‘supporting function’ in the person living with dementia? It is unsuitable to talk about ‘managing ‘ any person.

Page 44. Implications. Previously ‘speech-language pathologists’ is used, whereas ‘speech therapist ‘ is introduced in this section. Its better to use the same terminology throughout.

4. Punctuation - there are places where commas need to be included, as follows:

Page 17, line 247. Insert comma between reference [33] and 'memory book'.

Page 17, line 248. Insert comma between reference [30] and 'digital'.

5. Findings.

Page 9. Regarding reference to results of authors Moos et al [33] and some other author [14], it would be preferable to report these results with all other results in the most relevant place. Include the Author of ref [14] in this sentence.

Table 3. Remove all superfluous words to ensure the content is precise and consistently stated, e.g. for ref Moos et al., 2006. Colum titled 'Type' remove words 'This study' and any other descriptive statements. Just list the type of LSB approaches used. In column titled ‘Session Time/Frequency’, removal all unnecessary words and just list terms such as ‘Variable – daily-weekly’. In column titled Length of Time’ remove all unnecessary words and just list terms such as ‘Variable – 5 mins-1 hour’.

Table 4. Remove all superfluous words to ensure the content is precise and consistently stated. As well, us eth same tense throughout when reporting results, e.g. ‘Enhanced quality and quantity of verbal interaction’, ‘Improved communication’, ‘Staff-reported increased motivation in care and interactive communication’.

Table 4. Includes Author names, but not reference numbers. Claims are mage regarding changes for persons living with dementia such as improved psychological health, but this is not supported by the evidence. It appears that the staff or family perceptions are determined to be evidence of these improvements, so if this is the case it needs to be reported as such.

Page 37, line 346. Insert name of author before the ref ‘[38]’.

Page 42. Conclusions. Remove term ’significant’ and replace with a more suitable term to describe the findings, e.g. ‘Some positive outcomes for the person living with dementia are reported, although more robust research is needed to determine the effects of the life story book for this population’.
